# AIM2 promotes irradiation resistance, migration ability and PD-L1 expression through STAT1/NF-κB activation in oral squamous cell carcinoma

**DOI:** 10.1186/s12967-023-04825-w

**Published:** 2024-01-03

**Authors:** Hui-Wen Chiu, Hsin-Lun Lee, Hsun-Hua Lee, Hsiao-Wei Lu, Kent Yu-Hsien Lin, Yuan-Feng Lin, Che-Hsuan Lin

**Affiliations:** 1 Graduate Institute of Clinical Medicine, College of Medicine, Taipei Medical University, Taipei, 11031 Taiwan; 2 Department of Medical Research, Shuang Ho Hospital, Taipei Medical University, New Taipei City, 23561 Taiwan; 3 TMU Research Center of Urology and Kidney, Taipei Medical University, Taipei, 11031 Taiwan; 4 Department of Radiology, School of Medicine, College of Medicine, Taipei Medical University, Taipei, 11031 Taiwan; 5 Department of Radiation Oncology, Taipei Medical University Hospital, Taipei, 11031 Taiwan; 6 Department of Neurology, Taipei Medical University Hospital, Taipei Medical University, Taipei, 11031 Taiwan; 7 Department of Neurology, School of Medicine, College of Medicine, Taipei Medical University, Taipei, 11031 Taiwan; 8 Department of Neurology, Vertigo and Balance Impairment Center, Shuang Ho Hospital, Taipei Medical University, New Taipei City, 23561 Taiwan; 9 Department of Otolaryngology Head and Neck Surgery, Shuang Ho Hospital, Taipei Medical University, New Taipei City, 23561 Taiwan; 10 Department of Otolaryngology, Taipei Medical University Hospital, Taipei Medical University, Taipei, 11031 Taiwan; 11 Department of Obstetrics and Gynaecology, North Shore Private Hospital, Sydney, NSW Australia; 12 Department of Gynecology, Ryde Hospital, Northern Sydney Local Health District, Sydney, NSW Australia; 13 Northern Clinical School, Faculty of Medicine and Health, The University of Sydney, Sydney, NSW Australia; 14 Cell Physiology and Molecular Image Research Center, Wan Fang Hospital, Taipei Medical University, Taipei, 11696 Taiwan; 15 Department of Otolaryngology, School of Medicine, College of Medicine, Taipei Medical University, 250 Wu-Hsing Street, Taipei, 11031 Taiwan

**Keywords:** Radioresistance, Metastasis, PD-L1, AIM2, Immune checkpoint inhibitors, Oral squamous cell carcinoma

## Abstract

**Background:**

Radioresistance and lymph node metastasis are common phenotypes of refractory oral squamous cell carcinoma (OSCC). As a result, understanding the mechanism for radioresistance and metastatic progression is urgently needed for the precise management of refractory OSCC. Recently, immunotherapies, e.g. immune checkpoint inhibitors (ICIs), were employed to treat refractory OSCC; however, the lack of predictive biomarkers still limited their therapeutic effectiveness.

**Methods:**

The Cancer Genome Atlas (TCGA)/Gene Expression Omnibus (GEO) databases and RT-PCR analysis were used to determine absent in melanoma 2 (AIM2) expression in OSCC samples. Colony-forming assay and trans-well cultivation was established for estimating AIM2 function in modulating the irradiation resistance and migration ability of OSCC cells, respectively. RT-PCR, Western blot and flow-cytometric analyses were performed to examine AIM2 effects on the expression of programmed death-ligand 1 (PD-L1) expression. Luciferase-based reporter assay and site-directed mutagenesis were employed to determine the transcriptional regulatory activity of Signal Transducer and Activator of Transcription 1 (STAT1) and NF-κB towards the AIM2-triggered PD-L1 expression.

**Results:**

Here, we found that AIM2 is extensively upregulated in primary tumors compared to the normal adjacent tissues and acts as a poor prognostic marker in OSCC. AIM2 knockdown mitigated, but overexpression promoted, radioresistance, migration and PD-L1 expression via modulating the activity of STAT1/NF-κB in OSCC cell variants. AIM2 upregulation significantly predicted a favorable response in patients receiving ICI treatments.

**Conclusions:**

Our data unveil AIM2 as a critical factor for promoting radioresistance, metastasis and PD-L1 expression and as a potential biomarker for predicting ICI effectiveness on the refractory OSCC.

## Introduction

Oral squamous cell carcinoma (OSCC) belongs to head and neck squamous cell carcinoma and accounts for 92–95% of oral malignancies [1]. The risk factors for OSCC are smoking, alcohol, betel nut chewing, nutritional deficiencies, and human papillomavirus (HPV) infection. Surgery alone or in combination with adjuvant radiotherapy with or without chemotherapy is the primary regimen for OSCC currently [2]. Recently, immunotherapy was developed to combat OSCC with no curative surgical and radiotherapeutic/chemotherapeutic treatment options [3]. Nevertheless, radioresistance/chemoresistance and the lack of biomarkers to predict the therapeutic effectiveness of immunotherapy impacted the clinical management of OSCC. As a result, understanding the molecular mechanism for radioresistance/chemoresistance and identifying valuable biomarkers for predicting the efficacy of immunotherapy are urgently needed.

Absent in melanoma 2 (AIM2) is one of the inflammasome members and acts as an innate immune sensor for double-strand DNA released from the invading pathogens. The assembly of AIM2 inflammasome promotes the secretion of interleukin-1β (IL-1β) and IL-18 and the induction of cell death through pyroptosis [4, 5]. The oncogenic role of AIM2 remains controversial in different cancer types. AIM2 upregulation has been correlated with a good prognosis [6] and suppresses epithelial–mesenchymal transition (EMT) via modulating PI3K/Akt and inflammasome pathways in colorectal cancer [7, 8]. Similarly, AIM2 could mitigate tumor growth and metastatic progression by inhibiting the PI3K/Akt/mTOR pathway in gastric cancer [9] and osteosarcoma [10]. The loss of AIM2 also promoted hepatocarcinoma progression through activation of the mTOR-S6K1 pathway [11] and EMT process [12]. In contrast, AIM2 upregulation promoted cell growth, metastasis and immunosuppression through the inflammasome-dependent pathway and correlated with poor survival in non-small cell lung cancer [13, 14], melanoma [15] and triple-negative breast cancer [16]. Besides, AIM2 was found to trigger renal cell carcinoma progression and sunitinib resistance through FOXO3a-ACSL4 axis-regulated ferroptosis [17]. In OSCC, the increased levels of AIM2 have been correlated with tumor growth and lymph node metastasis [18, 19]; however, the molecular mechanism remains largely unknown.

Consequently, this study aimed to investigate the involvement of AIM2 in the molecular mechanism for irradiation resistance, metastatic progression and immunomodulation in OSCC. Here, we find that AIM2 upregulation is commonly detected in primary tumors compared to normal adjacent tissues and refers to a poor response to radiotherapy in OSCC patients. Moreover, the artificial alteration of AIM2 expression causally affected the irradiation resistance, migration ability and programmed death-ligand 1 (PD-L1) expression in OSCC cells. Computational simulation by the Gene Set Enrichment Analysis program and cell-based assays revealed that AIM2 upregulation reinforces the transcriptional regulatory activity of STAT1/NF-κB towards the PD-L1 gene and fosters the metastatic and radioresistant phenotypes in OSCC cells. Fortunately, the increased levels of AIM2 highly correlate with a favorable responsiveness to immune checkpoint inhibitors, which provides an opportunity to combat those refractory OSCC with AIM2 upregulation in future clinics.

## Materials and methods

### Collection of clinical data and samples

The clinical information and transcriptional profiling data of head and neck squamous cell carcinoma (HNSCC) patients derived from The Cancer Genome Atlas (TCGA) and OSCC patients derived from the GSE42743 dataset were obtained from UCSC Xena website (UCSC Xena. Available online: http://xena.ucsc.edu/welcome-to-ucsc-xena/) and the Gene Expression Omnibus (GEO) database on the NCBI website, respectively. Primary tumors and normal adjacent tissues of OSCC patients were provided by Taipei Medical University Biobank and collected with institutional review board approval (N202204007) and the Declaration of Helsinki.

### Cell culture

Oral cancer cell lines HSC2, HSC3, HSC4 and SAS were obtained from the Japanese Collection of Research Bioresources (JCRB) Cell Bank cultivated in Eagle's minimal essential medium supplemented with 10% fetal calf serum and 1% (w/v) Penicillin–Streptomycin at 37 °C with 5% CO2. All culture reagents were purchased from Gibco (Grand Island, NY, USA).

### Irradiation exposure

Cells (70% confluence) cultivated in 25-T flasks were exposed to 6 MV X-rays using a linear accelerator (Digital M Mevatron Accelerator, Siemens Medical Systems, CA, USA) at a 4 Gy/min dose rate. To ensure electronic equilibrium and full backscatter, a tissue-equivalent bolus (2 cm) was placed on top of and tissue-equivalent material (10 cm) was placed under the 25-T flasks, respectively.

### Colony formation assay

Cells (2 × 10^3^) without or with irradiation exposure at 4 Gy were seeded on the 6-well plates and cultured for two weeks. At the end of cultivation, the cells were fixed with 80% ethanol prior to crystal violet (0.1%) staining for 10 min. After several wash steps, the remaining crystal violet was solubilized by using 30% acetic acid and its optical density was measured at 595 nm wavelength by a photometer.

### Cell migration assay

Trans-well cultivation was performed by Boyden Chamber Assay (NeuroProbe, Gaithersburg, MD, USA) according to the procedure as shown in our previous report [14]. For neutralization of IL-1β, AIM2-overexpressing SAS cells (1.5 × 10^4^) were pre-treated without or with rabbit anti-IL-1β (#41,059, SAB, Greenbelt, MD, USA) and non-immunized control (#10,119-T52/#CR1, Sino Biological, Beijing, China) polyclonal antibodies at 10 μg/ml for 24 h before the trans-well culture for 16 h. Giemsa solution was used to stain the migrated cells after the removal of non-migrated cells. The stained cells were observed under an optical microscope and quantified in four random areas at 400 × magnification.

### Plasmid construction, lentiviral particle preparation and lentivirus infection

Human AIM2 cDNA clone (HG11654-UT) was purchased from Sino Biological Inc. (Beijing, China) and amplified by using the sticky-end polymerase chain reaction (PCR) method with two paired primers (primer #1: *CTAGC*ATGGAGAGTAAATACAAGGAGATACTCTTG and *C*CTATGT TTTTTTTTTGGCCTTAATAACC, primer #2: *C*ATGGAGAGTAAATACAAGGAGATACTCTTG and *AATTC*CTATGTTTTTTTTTTGGCCTTAATAACC) to generate the PCR products displaying cohesive ends compatible with NheI/EcoRI restriction sites. The sticky-end PCR products were the subclone into the lentiviral shuttle vector pLAS3w/puro using NheI/EcoRI restriction sites. The production of lentiviral particles containing without or with the AIM2 gene was performed through collaboration with the National RNAi Core Facility at Academia Sinica in Taiwan. The pLKO.1/puro plasmid containing non-silencing control (oligo sequence: CCGGACACTCGAGCACTTTTTG) and AIM2 shRNA clones (target sequence: sh1_CCCGCTGAACATTATCAGAAA; sh2_CCAACTGGTCTAA GCAGCATT) packaged in lentiviral particles were also obtained from the National RNAi Core Facility. Cells with 50% confluence cultivated in the 6-well plates were incubated with the conditioned medium containing polybrene (Santa Cruz) at 5 μg/ml and then infected with the lentiviral particles containing without (non-silencing or vector control) or with AIM2 shRNAs or exogenous AIM2 gene at a multiplicity of infection (MOI) of 3–10. Puromycin (10 μg/ml) selection was finally performed to generate stable clones. Western blot analysis and reverse transcription-polymerase chain reaction (RT-PCR) were used to confirm AIM2 knockdown and overexpression efficiency in the puromycin-resistant cells.

### RT-PCR

Total RNA was extracted by TRIzol extraction kit (Invitrogen) from the tested cells. M-MLV reverse transcriptase (Invitrogen) was employed for converting total RNA (5 μg) to cDNA which was then amplified by PCR method using Taq-polymerase (Protech) with paired primers (for AIM2, forward-CTGCACCAAAAGTCTCTCCTCATG and reverse-GGCTGAGTTTGAAGCGTGTTGAT C; for PD-L1, forward-GCTGCACTTCAGATCACAGATGTG and reverse-GTGTTGATTCTCAGT GTGCTGGTC; for GAPDH, forward-AGGTCGGAGTCAACGGATTTG and reverse-GTGATGGC ATGGACTGTGGTC).

### Western blot and dot blot assays

For Western blot analysis, aliquots of cell lysate (20–100 μg) were subjected to SDS gel electrophoresis and then transferred to PVDF membranes. For dot blot analysis, culture media (200 μL) were loaded into the well of dot blot apparatus and then transferred to nitrocellulose membrane by suction. The membranes were then immersed in the blocking buffer [5% bovine serum albumin for Western blot analysis against the phosphorylated protein and dot blot analysis or 5% skim milk in Tris buffer saline containing 0.1% Tween-20 (TBST)] for 2 h at room temperature or overnight at 4 °C with a gentle agitation. After blocking step, the membranes were further incubated with primary antibodies against PD-L1 (GTX635975, GeneTex, Hsinchu, Taiwan), phosphorylated (Ser536) NF-κB (#3033)/NF-κB (#6956)/phosphorylated (Tyr701) STAT1 (#9167)/STAT1 (#9172) (Cell Signaling, Danvers, MA, USA) and GAPDH (#PA0212, AbFrontier, Seoul, Korea), IL-1β (#41,059, SAB) overnight at 4 °C. After the incubation, the membranes were washed several times with TBST followed by another incubation with a horseradish peroxidase-labeled secondary antibody for 1 h at room temperature. Immunoblots were then visualized by an enhanced chemiluminescence system (Amersham Biosciences, Tokyo, Japan).

### Flow-cytometric analysis

Cells (1 × 10^7^) from the designed experiments were collected by using dislodging solution (0.15 g disodium EDTA, 4.0 g NaCl, 0.28 g sodium bicarbonate, 0.5 g dextrose and 0.2 g KCl dissolved in 500 ml double distilled water and filter-sterilized) and stained with Brilliant Violet 605™ anti-human CD274 (PD-L1, #329,706, Biolegend, San Diego, CA, USA) antibody and its isotype IgG control antibody (2 μl of each) for 20 min. After several washes with phosphate-buffered saline, the stained cells were subjected to Flow-cytometric analysis.

### Enzyme-linked immunosorbent assay (ELISA)

The secreted IL-1β protein levels in the OSCC cell variant were detected by the commercial ELISA kits (Quantikine^®^ ELISA, R&D Systems, Minneapolis, MN, USA) according to the manufacturer’s protocol.

### Luciferase reporter assay and site-directed mutagenesis

Firefly luciferase reporter vectors harboring an interferon-gamma (IFNγ) activation site (GAS), pGL4[Luc2P/GAS-RE/Hygro] and NF-κB-binding element, pGL4.32[luc2P/NF-kB-RE/Hygro] were purchased from Promega (Madison, WI, USA), and containing PD-L1 promoter region (− 1 to 1000 bp) was obtained from Addgene (Watertown, MA, USA). Site-directed mutagenesis for GAS and NF-κB-binding element within PD-L1 promoter was performed by PCR with the paired primers (for GAS, 1st run_forward-CCATATGGCTTTGGTTTTTATTCTAAGGAGTAGGTGTGTGTGTGT GTGTATGG and reverse-CCATACACACACACACACACCTACTCCTTAGAATAAAAACCAAA GCCATATGG, 2nd run_ forward-GACCCATATGGCTTTGGTTTTTACCTTAAGGAGTAGGTGT GTGTGTGTG and reverse-CACACACACACACCTACTCCTTAAGGTAAAAACCAAAGCCAT ATGGGTC; for NF-κB-binding element, 1st run_forward-CTTCATTTGCTTTGTCATTAAAAGAA CTGAAAATCCCGAGCTACATCTTTTAAGAATGCTCAG and reverse-CTGAGCATTCTTAAA AGATGTAGCTCGGGATTTTCAGTTCTTTTAATGACAAAGCAAATGAAG, 2nd run_ forward-ATAATGAAACTTCATTTGCTTTGTCATTAAAAATTCTGAAAATCCCGAGCTACATCTTTTAAGAATG and reverse-CATTCTTAAAAGATGTAGCTCGGGATTTTCAGAATTTTTAATGACAA AGCAAATGAAGTTTCATTAT) using a *pfu* DNA polymerase kit (Stratagene, La Jolla, CA, USA). The methylated parental DNA template was removed by the treatment with *Dpn1* endonuclease (New England BioLabs, Hitchin, Hertfordshire, UK). Luciferase reporter assay was performed by co-transfecting cells (70% confluence) with the designed firefly luciferase reporter vector at 0.25 μg and control *Renilla* luciferase-expressing vector (p4.74-RLuc, Promega) at 0.0125 μg using Lipofectamine 2000 (Invitrogen, Thermo Fisher Scientific, Waltham, MA, USA) according to the manufacturer’s protocol. Post-transfection for 24 h, cells were lysed in Dual-Glo™ Luciferase reagent. Luciferase activities were detected by Dual-Glo^®^ Luciferase Assay (Promega) according to the manufacturer’s protocol. The luminescent intensities of firefly luciferase derived from the tested cells were normalized to that of co-transfected *Renilla* luciferase.

### Kaplan–Meier analysis

The Kaplan–Meier survival curves were generated from the K–M Plotter website according to the method reported by Lanczky A and Gyorffy B [20]. The best cutoff values, stratifying patients into the low and high-expression groups, were determined by the highest hazard ratio in the Cox regression test and the lowest false discovery rate (FDR) in the Benjamini–Hochberg test.

### Gene Set Enrichment Analysis (GSEA)

GSEA software (version 4.0.3) was downloaded from Molecular Signature Database (MSigDB, https://www.gsea-msigdb.org/gsea/index.jsp). We then determined whether priori-defined Hallmark gene sets from MSigDB were enriched in the AIM2-related gene signature generated by pre-ranking the somatic genes which were included in the GSE42743 microarray experiment with Pearson coefficient values from the tests of their co-expression with AIM2 gene in primary tumors of GSE42743 OSCC patients who received radiotherapy and was recorded as dead in the follow-up. The AIM2-related gene signature was analyzed by the GSEA Preranked module with recommended default settings (1000 permutations and a classic scoring scheme). The enrichment score (ES), normalized enrichment score (NES), nominal p-value and FDR q-value were calculated by the methods reported by Aravind Subramanian et al. [21] and obtained from the GSEA report. The p and q values lower than 0.05 were considered as significant.

### Analysis of tumor-infiltrating immune cells

TIMER2.0, a web server (http://timer.cistrome.org/), reported by Taiwen Li et al. [22] was used to estimate the correlation among AIM2 mRNA levels, tumor purity and immune cell infiltration levels in TCGA HNSCC samples. The TIME2.0 reports shown as a scatter plot were downloaded from the website.

### Statistical analysis

SPSS Statistics 19 software (IBM, Armonk, NY, USA) was used to analyze statistical significance. Student t-test was used to estimate the statistical difference of AIM2 expression in primary tumors and normal tissues. The correlation among AIM2 mRNA levels, immune cell infiltration levels and other somatic gene expression levels were analyzed by Pearson’s and nonparametric Spearman’s tests in the detected primary tumors. Nonparametric Mann–Whitney U and Friedman tests were employed to estimate the two independent samples and three or more related samples, respectively. The *p* values lower than 0.05 were considered to be statistically significant in all experiments.

## Results

### AIM2 upregulation is extensively detected in primary tumors compared to normal adjacent tissues and correlates with poor sensitivity to radiotherapy in OSCC

**Fig. 1:**
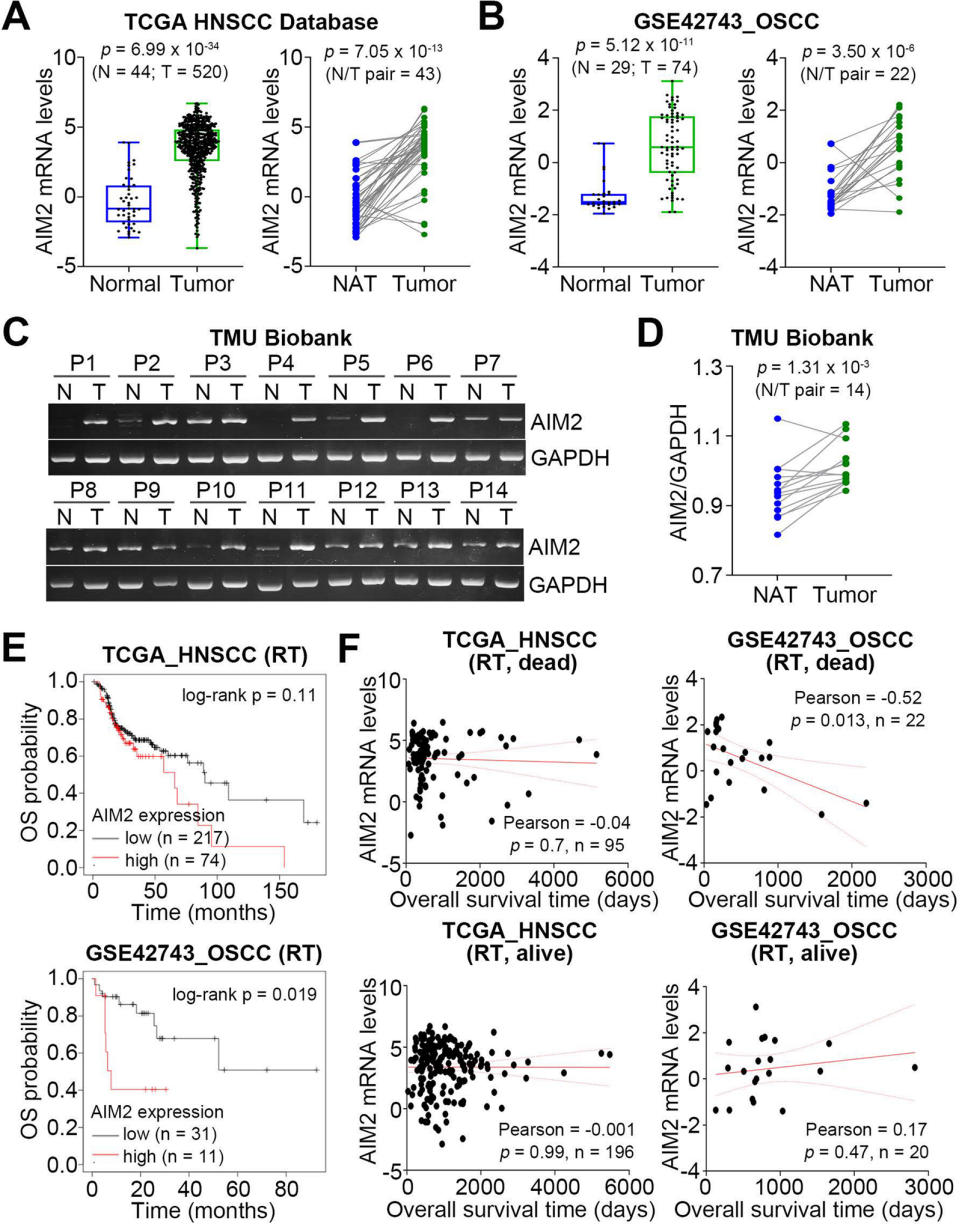
AIM2 upregulation is highly detected in primary tumors compared to normal adjacent tissues and correlates with a poor response to radiotherapy in OSCC. **A** and **B** Boxplots and dot plots with line respectively present AIM2 mRNA levels in the unpaired normal tissues (N)/primary tumors (T) and paired normal adjacent tissues (NAT)/primary tumors derived from TCGA HNSCC (**A**) and GSE4273 OSCC (**B**) samples. **C** and **D** RT-PCR (**C**) and dot plots with lines (**D**) show the AIM2 gene expression in the paired primary tumors/normal adjacent tissues derived from OSCC samples in Taipei Medical University (TMU) Biobank. **E** Kaplan–Meier analyses for AIM2 mRNA levels using overall survival (OS) probability under a maximal risk condition against TCGA HNSCC (upper) and GSE42743 OSCC (lower) patients who received radiotherapy. **F** Scatchard plots for the correlation between AIM2 mRNA levels in the respective primary tumors and overall survival time of TCGA HNSCC and GSE42743 OSCC patients receiving radiotherapy (RT) with the overall survival endpoint as alive or dead

We dissected the transcriptional profile of AIM2 in the normal tissues and primary tumors derived from head and neck squamous cell carcinoma (HNSCC) patients in The Cancer Genome Atlas (TCGA) database. The data showed that AIM2 expression in primary tumors is extensively higher than in normal tissues (Fig. 1A). Similar views were found in the paired normal and tumor tissues derived from TCGA HNSCC patients (Fig. 1A). Using the GSE42743 dataset, we also found that AIM2 upregulation is predominant in primary tumors compared to normal tissues or adjacent normal tissues from OSCC patients (Fig. 1B). Accordingly, RT-PCR results demonstrated that AIM2 expression is significantly (p < 0.01) elevated in primary tumors compared to the normal adjacent tissues from OSCC patients in Taipei Medical University Biobank (Fig. 1C and D). Kaplan–Meier analyses using a maximal risk condition demonstrated that AIM2 upregulation significantly (p = 0.019) was associated with a poorer overall survival in GSE42743 OSCC, but not TCGA HNSCC, patients who received radiotherapy (Fig. 1E). Accordingly, AIM2 upregulation was significantly (p = 0.013) correlated with a shorter overall survival time in GSE42743 OSCC patients who received radiotherapy and were recorded dead in the follow-up (Fig. 1F). However, there were no statistical significances in the correlation between AIM2 expression and overall survival time in the TCGA HNSCC cohorts who received radiotherapy regardless of survival status and GSE42743 OSCC patients who received radiotherapy and were recorded as alive in the follow-up (Fig. 1F).

### AIM2 upregulation promotes radioresistance and metastatic potentials in OSCC cells

**Fig. 2:**
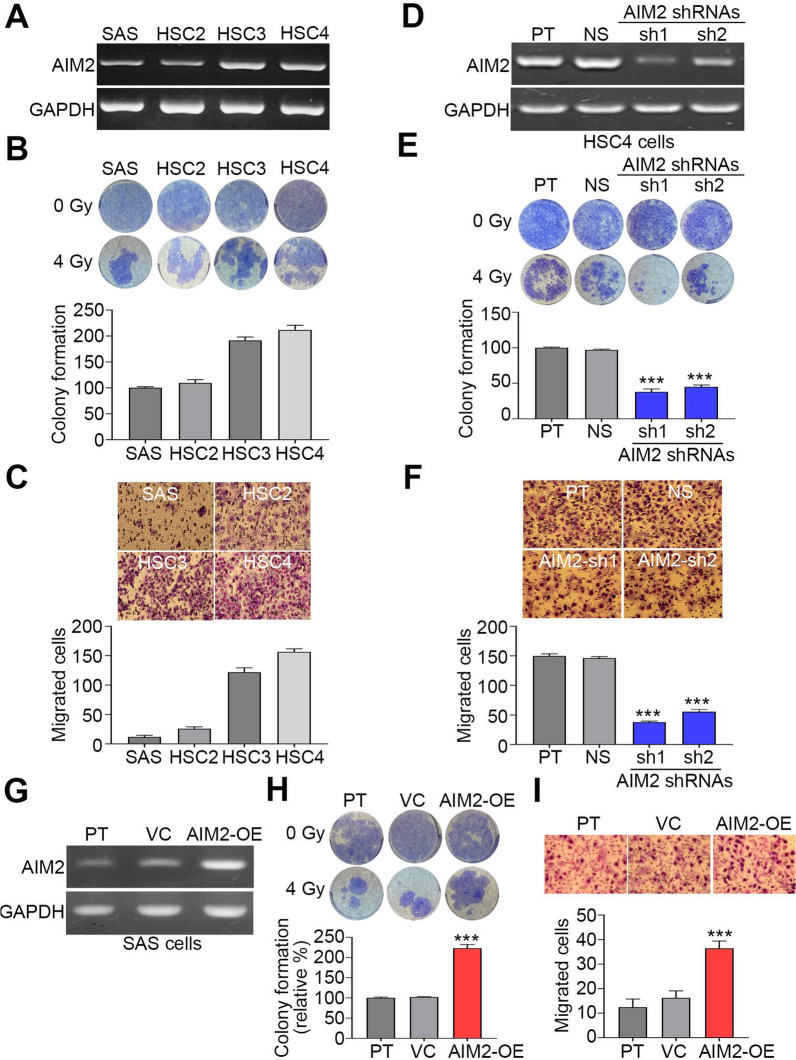
AIM2 expression is associated with cellular irradiation resistance and migration ability in OSCC cells. **A**–**I** RT-PCR for the endogenous mRNA levels of AIM2 and GAPDH, crystal violet staining (upper)/histogram (lower) for colony formation post-treatment without or with irradiation at 4 Gy and Giemsa staining (upper)/histogram (lower) for the migrated cells in the tested OSCC cell lines (**A**–**C**), HSC4 cell variants (**D**–**F**) and SAS cell variants (**G**–**I**). in **A**, **D** and **G**, GAPDH was used as an internal control of the experiment. The abbreviations of PT, NS, VC and OE are parental, non-silencing, vector control and overexpression, respectively. ***p < 0.001

We next determined the endogenous mRNA levels of AIM2 by RT-PCR experiment in a panel of OSCC cell lines SAS, HSC2, HSC3 and HSC4. The data showed that HSC3 and HSC4 cells exhibit a relatively higher mRNA level of AIM2 than SAS and HSC2 cells (Fig. 2A). The endogenous mRNA levels of AIM2 were found to negatively associate with the cytotoxicity of irradiation (Fig. 2B) but positively correlate with cellular migration ability (Fig. 2C) in the tested OSCC cell lines. The knockdown of the AIM2 gene in HSC4 cells (Fig. 2D) dramatically restored the cellular sensitivity to irradiation treatment (Fig. 2E) and predominantly mitigated the cellular migration ability (Fig. 2F). Conversely, the enforced expression of the exogenous AIM2 gene in SAS cells (Fig. 2G) robustly promoted radioresistance (Fig. 2H) and markedly enhanced the cellular migration ability (Fig. 2I).

### AIM2 upregulation activates STAT1 and NF-κB-related signaling cascades to potentiate PD-L1 expression in OSCC cells

**Fig. 3:**
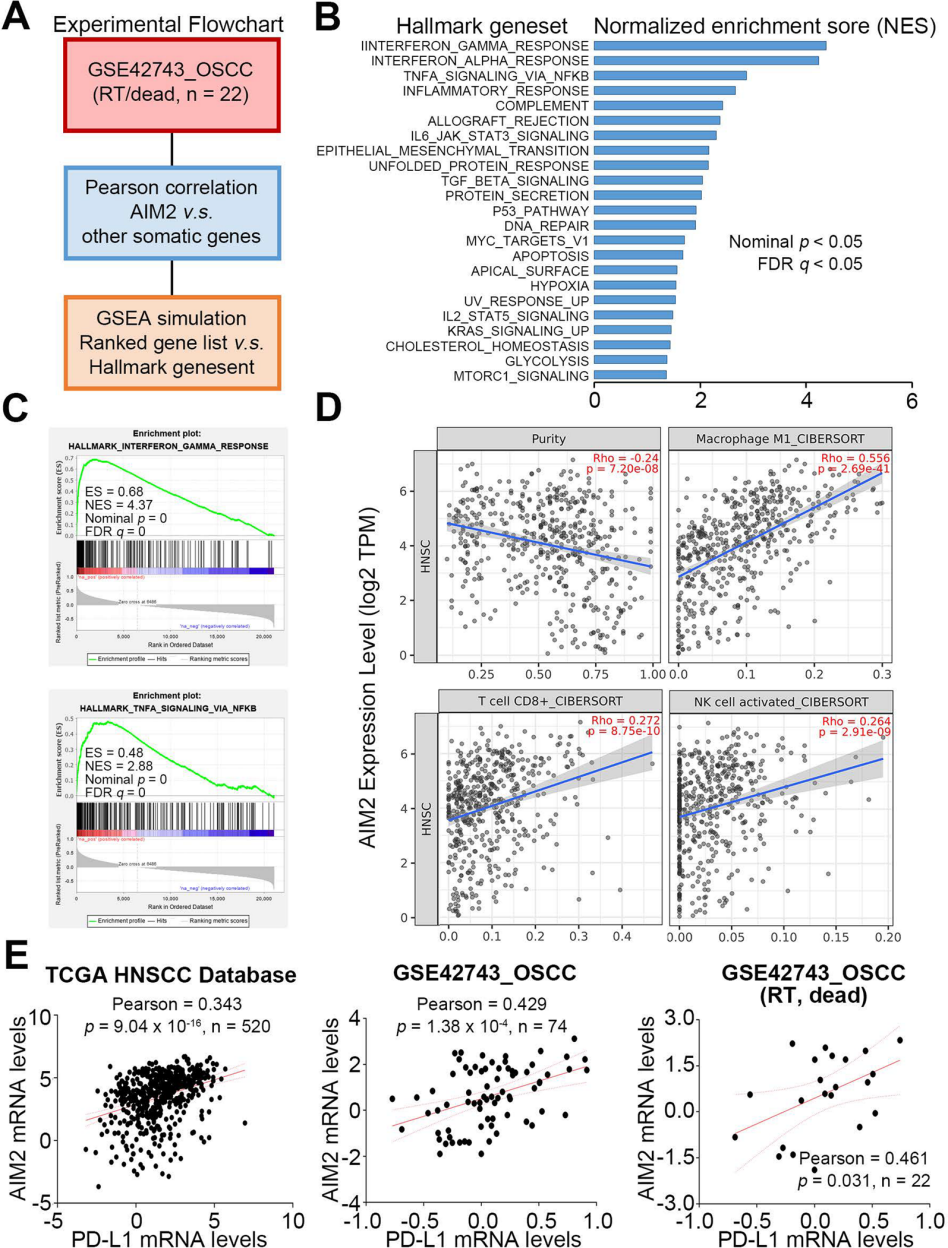
AIM2 upregulation highly correlates with the activation of interferon and inflammation-related pathways and the expression of PD-L1 in the primary tumors derived from OSCC patients with a poorer responsiveness to radiotherapy. **A** Experimental flowchart for generating AIM2 gene signature and performing GSEA simulation. **B** Histogram for Hallmark genesets with positive NES and statistical significance (*p* and *q* < 0.05) in GSEA simulation against AIM2 gene signature. **C** The plot of enrichment score for IFNγ-responsive and TNFα/NF-κB signaling axis-regulated genesets in GSEA simulation against AIM2 gene signature. **D** Scatchard plot derived from TIMER2.0 for the correlation of AIM2 mRNA levels with tumor purity and infiltration levels of macrophages, CD8 + T cells and NK cells against TCGA HNSCC. **E** Scatchard plot for the co-expression of AIM2 and PD-L1 in TCGA HNSCC primary tumors (left) and GSE42743 OSCC samples derived from patients without (middle) or with (right) the indicated records. In **D** and **E**, the solid and dashed lines shown in red represent the 95% confidence bands (dashed) of the best-fit line (solid) in Simple Linear Regression

To understand the possible mechanism underlying the AIM2-associated radioresistance in OSCC, Gene Set Enrichment Analysis (GSEA) was conducted to detect whether a series of priori-defined biological processes deposited in Hallmark gene sets from the Molecular Signature database were enriched in the gene rank of AIM2-related gene signature from primary tumors of GSE42743 OSCC patients who received radiotherapy and was recorded as dead in the follow-up. The Pearson coefficient values derived from the correlation among the mRNA levels of AIM2 and somatic genes included in the microarray chip of the GSE42743 dataset were used to generate a ranked order of somatic genes, which was defined as the AIM2-related gene signature (Fig. 3A). GSEA results indicated that the AIM2-related gene signature is highly correlated with the upregulation of interferon-gamma (IFNγ) responsive gene set, as well as TNFα-NF-κB signaling axis-regulated gene set (Fig. 3B and C). By using the TIMER2.0 program, we found that AIM2 mRNA levels positively correlate with the infiltration levels of IFNγ-producing CD8 + T cells and nature killer (NK) cells and TNFα-secreting M1 macrophages in TCGA HNSCC samples (Fig. 3D). Since programmed death-ligand 1 (PD-L1) is one of the IFNγ-inducing genes, we further examined the co-expression of AIM2 and PD-L1 in TCGA HNSCC and GSE42743 OSCC samples. The data showed that AIM2 expression was positively associated with PD-L1 expression in TCGA HNSCC and GSE42743 OSCC samples (Fig. 3E). This positive correlation was more predominant in the GSE42743 OSCC samples derived from patients who received radiotherapy and were recorded dead in the follow-up (Fig. 3E).

**Fig. 4:**
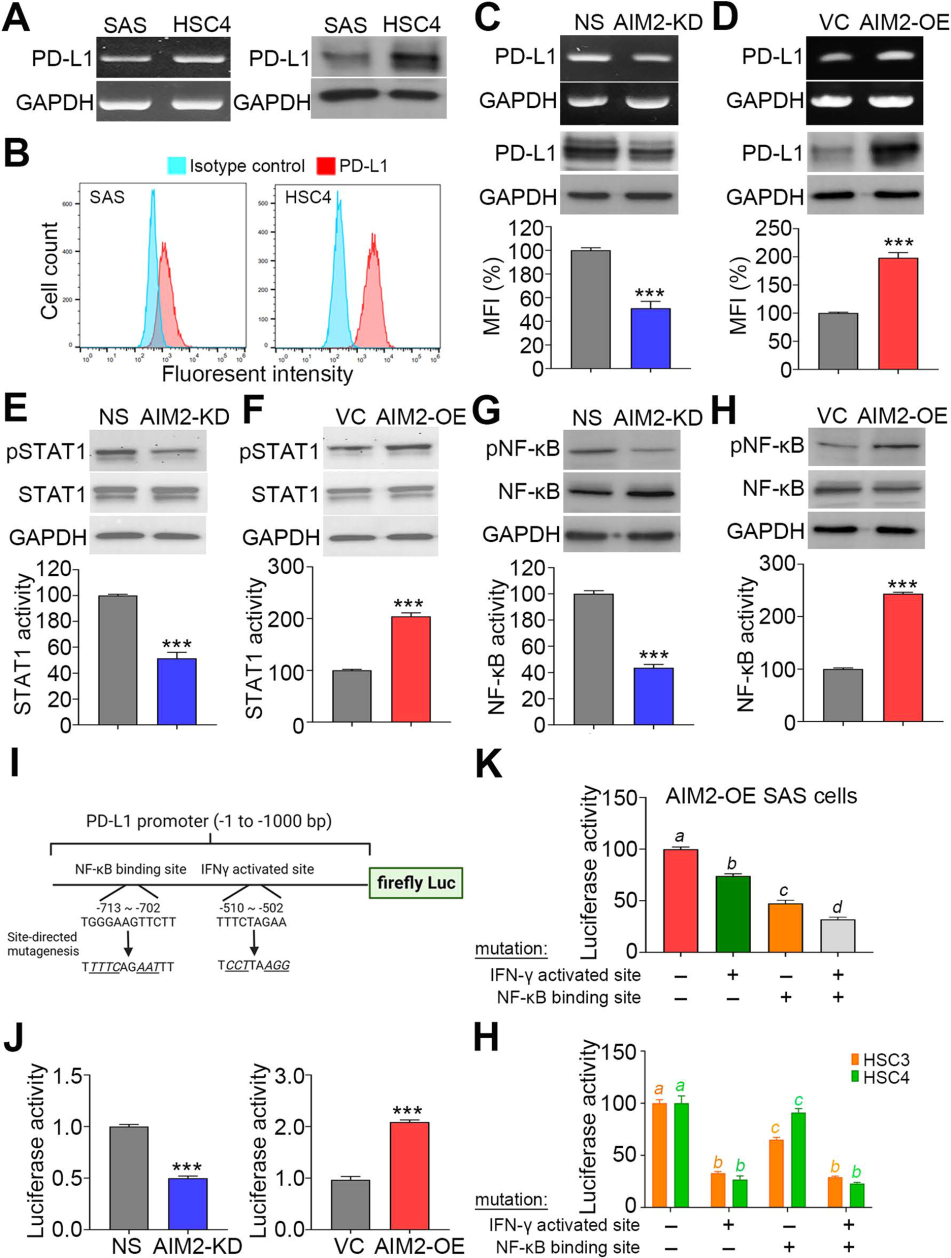
AIM2 upregulation promotes PD-L1 expression via activating STAT1 and NF-κB signaling pathways in OSCC cells. **A** and **B** RT-PCR (left)/Western blot analyses (right) for the endogenous mRNA/protein levels of PD-L1 and GAPDH (**A**) and histograms for the mean of fluorescent intensity (MFI) of isotype control (cyan) and PD-L1 (pink) antibodies in Flow-cytometric analysis (**B**) against SAS and HSC4 cells. **C** and **D** RT-PCR (upper)/Western blot analysis(middle) for the endogenous mRNA/protein levels of PD-L1 and GAPDH and histograms for the MFI of PD-L1 antibody normalized to control groups (NS and VC) in Flow-cytometric analysis against HSC4 (**C**) and SAS (**D**) cell variants. **E**–**H** Western blot analyses for phosphorylated STAT1/NF-κB, total STAT1/NF-κB and GAPDH protein levels (upper) and histograms for the DNA-binding activity of STAT1/NF-κB normalized to control groups (NS and VC) in luciferase reporter assay (lower) against HSC4 (**E** and **G**) and SAS (**F** and **H**) cell variants. **I** Illustration for the locations and mutated nucleotides of NF-κB binding site/IFNγ activated site in the PD-L1 promoter region subcloned into the upstream of the firefly luciferase gene. **J**–**H** Histograms for the normalized luciferase activity detected from HSC4/SAS cell variants (**J**), AIM2-overexpressing SAS cells (**K**) and HSC3/HSC4 cells (**H**) cells transfected with the PD-L1 promoter-driven firefly luciferase reporter vector without or with mutations at NF-κB binding site and IFNγ activated site. In **A**, **C**, **D**, **E**, **F** and **G**, GAPDH was used as an internal control of experiments. The symbol “***” and different alphabets denote *p* < 0.001 and 0.05, respectively

We further determined the endogenous PD-L1 levels in the radiosensitive/poorly migrated SAS cells and radioresistant/highly migrated HSC4 cells. The data obtained from RT-PCR/Western blot analyses (Fig. 4A) and flow-cytometric analysis (Fig. 4B) revealed that HSC4 cells, compared to SAS cells, harbor higher endogenous mRNA/protein levels and membranous protein levels of PD-L1. Whereas AIM2 knockdown in HSC4 cells markedly inhibited PD-L1 expression (Fig. 4C), AIM2 overexpression predominantly promoted PD-L1 levels in SAS cells (Fig. 4D). To delineate if AIM2 expression can affect the activity of IFNγ-STAT1 and TNFα-NF-κB signaling pathways, we performed another Western blot analysis to examine the actively phosphorylated protein levels of STAT1 and NF-κB and luciferase reporter assays to determine the DNA-binding activity of STAT1 and NF-κB. The data showed that AIM2 knockdown in HSC4 suppresses, but AIM2 overexpression in SAS cells elevates the phosphorylated protein levels and the transcriptional regulator activity of STAT1 (Fig. 4E and F) and NF-κB (Fig. 4G and H). To understand the transcriptional regulation of STAT1 and NF-κB towards the PD-L1 gene, we further established the luciferase-based promoter assay by sub-cloning PD-L1 promoter sequence (− 1 to − 1000 bp) into the upstream of the firefly luciferase gene (Fig. 4I). According to the previous report [23], we found a putative IFNγ-activated site (− 502 to − 510) which facilitates the binding of active STAT1 within the PD-L1 promoter sequence (Fig. 4I). Moreover, the in silico analysis by the PREMO program indicated a putative NF-κB binding site (− 502 to − 510) in the targeted PD-L1 promoter sequence (Fig. 4I). Whereas AIM2 knockdown in HSC4 cells was dramatically suppressed, AIM2 overexpression in SAS cells predominantly enhanced the intracellular luciferase activities after the transfection with the plasmid containing a PD-L1 promoter-driven firefly luciferase gene (Fig. 4J). Furthermore, we performed the site-directed mutagenesis for IFNγ-activated site and NF-κB binding site to verify the functional role of STAT1 and NF-κB in regulating the transcription of the PD-L1 gene (Fig. 4I). Luciferase reporter assay by transfecting AIM2-overexpressing SAS cells with the firefly luciferase gene in conjunction with the different PD-L1 promoter variants demonstrated that the mutation of IFNγ activated site or/and NF-κB binding site significantly (*p* < 0.05) reduced the intracellular luciferase activity (Fig. 4K). Similar views were also found in the AIM2-upregulated HSC3/HSC4 cells transfected with the firefly luciferase gene harboring the different PD-L1 promoter variants (Fig. 4H). These findings indicate that AIM2 upregulation fosters the transcription of PD-L1 by activating STAT1 and NF-κB in the metastatic/radioresistant OSCC.

### AIM2 upregulation promotes the secretion of IL-1β, which activates STAT1/NF-κB-related pathways to reinforce cellular irradiation resistance, migration ability and PD-L1 expression in OSCC cells

**Fig. 5:**
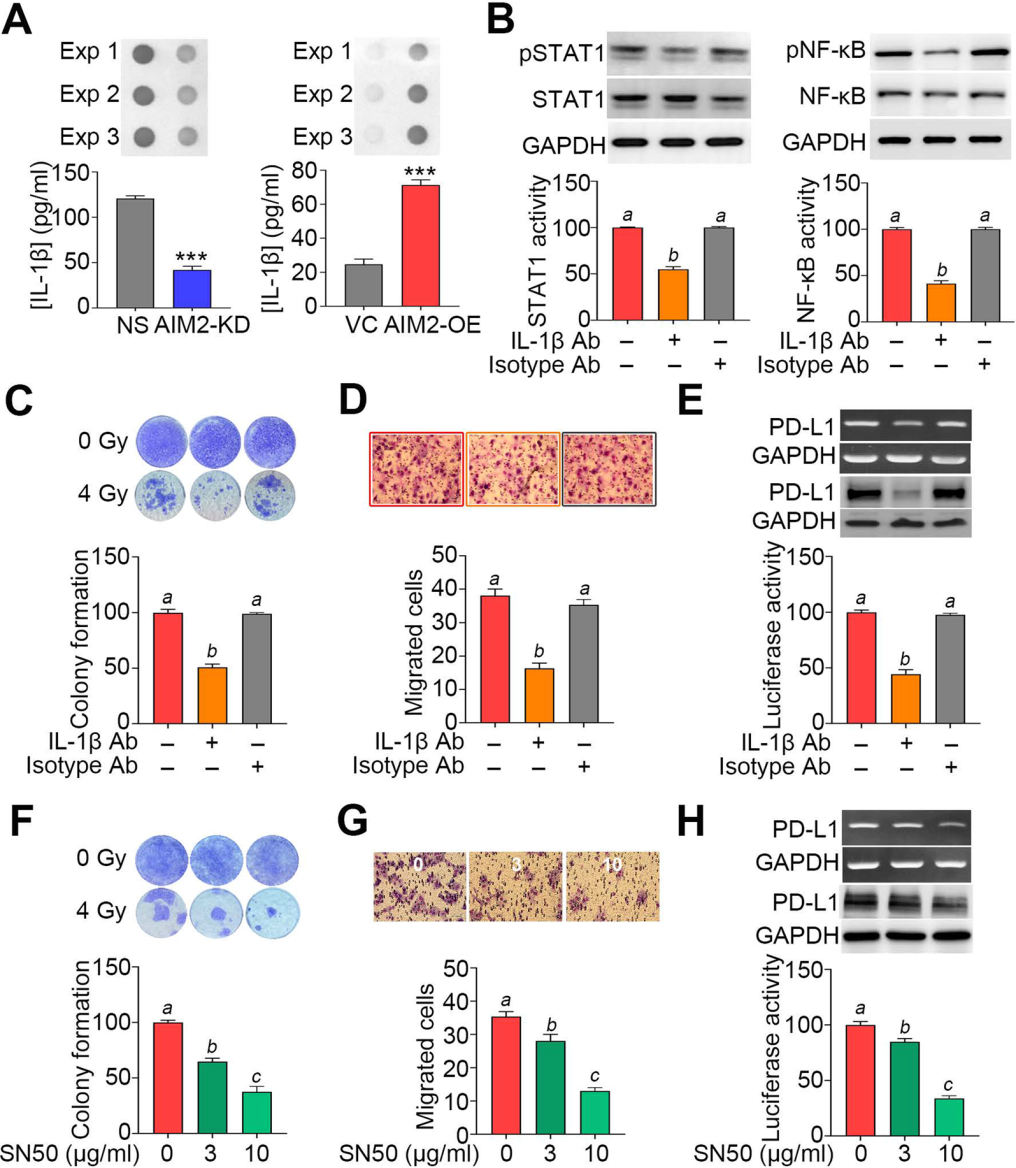
The AIM2-triggered IL-1β secretion positively regulates STAT1/NF-κB activation, irradiation resistance, cellular migration ability and PD-L1 expression in OSCC cells. **A** Dot blot (upper) of three independent experiments (Exp) and histograms (lower) of ELISA results for the IL-1β protein levels in the culture media from HSC4 (left) and SAS (right) cell variants. **B** Western blot analyses for phosphorylated STAT1/NF-κB, total STAT1/NF-κB and GAPDH protein levels (upper) and histograms for DNA-binding activity of STAT1/NF-κB in luciferase reporter assays (lower) against the AIM2-overexpressing SAS cells treated without or with IL-1β-neutralizing or isotype antibody (Ab, 10 μg/ml of each). **C**–**H** Crystal violet staining (upper)/histogram (lower) for colony formation post-treatment without or with irradiation at 4 Gy, Giemsa staining (upper)/histogram (lower) for the migrated cells and RT-PCR/Western blot analyses for the mRNA/protein levels of PD-L1 and GAPDH (upper)/histograms for PD-L1 transcriptional activity in luciferase assays (lower) against the AIM2-overexpressing SAS cells cultivated in the absence or presence of IL-1β-neutralizing/isotype antibody (**C**–**E**) and NF-κB inhibitor SN50 at the indicated concentrations (**F**–**H**). In **B**, **E** and **H**, GAPDH was used as an internal control of experiments. The symbol “***” and different alphabets represent p < 0.001 and p < 0.05, respectively

Interleukin-1β (IL-1β) secretion to the extracellular matrix is tightly regulated by inflammasome machinery. As a result, we next determined the extracellular protein levels of IL-1β by dot blot analysis and a commercial ELISA kit. The data showed that AIM2 knockdown in HSC4 cells markedly reduces, but AIM2 overexpression in SAS cells predominantly elevates the secretion of IL-1β (Fig. 5A). The inclusion of an IL-1β-neutralizing antibody, not an isotype control antibody, significantly (p < 0.05) suppressed STAT1/NF-κB activity, colony formation post-treatment with irradiation, cellular migration ability and PD-L1 expression in the AIM2-overexpressing SAS cells (Fig. 5B–E). Moreover, the pharmaceutical inhibition of NF-κB by its specific inhibitor SN-50 dose-dependently mitigated colony formation post-treatment with irradiation, cellular migration ability and PD-L1 expression in the AIM2-overexpressing SAS cells (Fig. 5F–H).

### AIM2 upregulation might be a valuable biomarker for predicting the anti-cancer effectiveness of immune checkpoint inhibitors on the metastatic/radioresistant OSCC

**Fig. 6:**
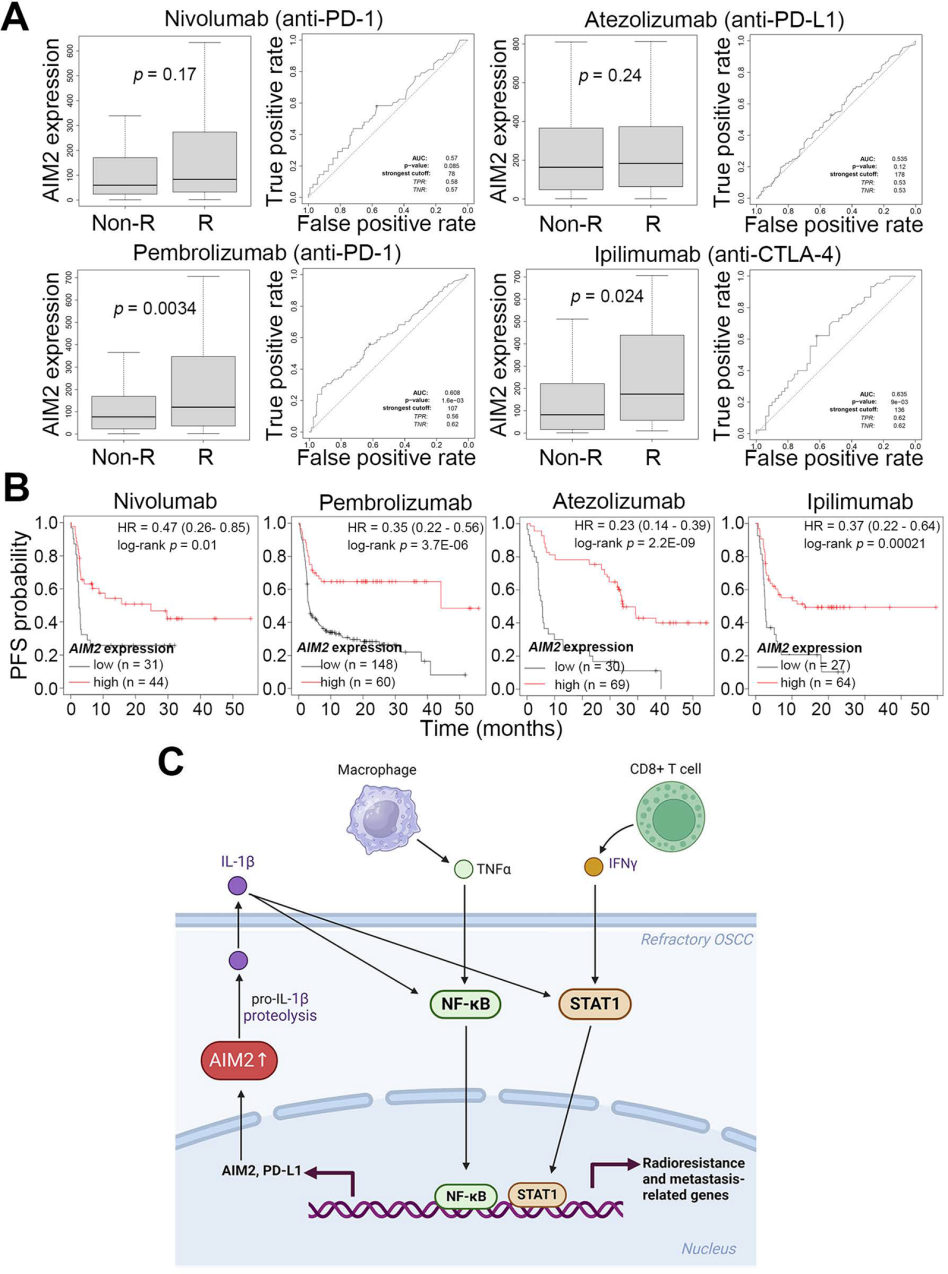
AIM2 upregulation correlates with a favorable response in cancer patients receiving immune checkpoint inhibitors. **A** Box plots for AIM2 mRNA levels in the non-responder (non-R) and responder (R) and receiver operating characteristic (ROC) curve (right) for the predictive sensitivity of AIM2 expression in ROC Plotter cancer patients receiving the indicated ICIs. **B** Kaplan–Meier analyses for AIM2 mRNA levels using progression-free survival probability under a maximal risk condition against K-M Plotter cancer patients receiving the indicated ICIs. **C** A proposed model for the AIM2-promoted radioresistance, metastasis and PD-L1 expression in refractory OSCC

The data obtained from the ROC plotter demonstrated that AIM2 upregulation predicts a favorable response to immune checkpoint inhibitors (ICIs), particularly in patients receiving Pembrolizumab (anti-PD-1 antibody) and Ipilimumab (anti-CTL4 antibody) (Fig. 6A). Although the expression of AIM2 in the primary tumors derived from non-responder and responder patients who received Nivolumab (anti-PD-1 antibody) and Atezolizumab (anti-PD-L1 antibody) treatment did not show a significant difference in ROC Plotter analysis (Fig. 6A), Kaplan–Meier plots under a maximal risk condition revealed that a higher level of AIM2 expression predominantly correlates with a good progression-free survival rate in cancer patients receiving these four ICIs (Fig. 6B).

## Discussion

The inflammasome activation was thought to be involved in the tumor progression of radioresistant breast cancer [24]. The recent report indicated that SP1 transcriptionally activates NLRP6 inflammasome to trigger immune evasion and radioresistance in glioma cells [25]. AIM2 upregulation was found to predict a favorable prognosis in colorectal cancer [7, 8], gastric cancer [9] and osteosarcoma [10], but associated with cancer progression, e.g. tumor growth, metastasis and immunosuppression, in non-small cell lung cancer [13, 14], melanoma [15], triple-negative breast cancer [16] and renal cell carcinoma [17]. AIM2 upregulation due to promoter hypomethylation has been shown to play an oncogenic role [26] and the enforced expression of AIM2 promoted tumor growth and lymph node metastasis in OSCC [18, 19]. However, the molecular mechanism for the AIM2-promoted OSCC progression remains largely unknown. Here, we further show that AIM2 upregulation promotes radioresistance, metastasis and PD-L1 expression, probably through an IL-1β-triggered activation of STAT1 and NF-κB pathways in refractory OSCC cells (Fig. 6C). The in silico analyses demonstrate that AIM2 upregulation fosters the infiltration of IFNγ-producing CD8 + T cells and TNFα-secreting macrophages to reinforce the activation of IFNγ-STAT1 and TNFα-NF-κB signaling pathways, which may ultimately force the expression of AIM2 and PD-L1, both are IFNγ-inducing genes [27, 28], in refractory OSCC cells (Fig. 6C). The formation of this signaling loop consequently potentiates the malignancy, e.g., radioresistance, metastasis and immune evasion, of OSCC. Fortunately, our findings provide a potential strategy to combat these refractory OSCC with AIM2 upregulation by immune checkpoint inhibitors Pembrolizumab and Ipilimumab.

NF-κB activation was considered a critical trigger for radioresistance [29]. As a result, targeting NF-κB activity was thought to be a potential strategy to combat cancer radioresistance [30]. Recent reports demonstrated that the activation of the TNF-α/NF-κB signaling axis enhances the metastatic capacity and PD-L1 expression of OSCC [31, 32]. Inhibition of NF-κB was found to mitigate the cellular invasive ability and restore the responsiveness to radiotherapy in glioblastoma [33]. In this study, GSEA simulation indicated that AIM2 upregulation highly correlates with the activation of TNF-α/NF-κB pathway in OSCC tumors derived from patients recorded as dead in the follow-up after irradiation treatment. Besides, AIM2 overexpression promoted the pro-inflammatory cytokine IL-1β which further reinforces the activation of the NF-κB pathway in SAS cells. The blocking of the NF-κB pathway restored the radiosensitivity and suppressed the cellular migration ability and PD-L1 expression in the AIM2-overexpressed SAS cells. Importantly, the activation of NF-κB may also enhance the transcription of other pro-inflammatory factors, e.g., T cell/macrophage chemotaxis cytokine CXCL10 [34], and thereby promote the infiltration of those immune cells to the tumor microenvironment and reinforce the function of IFNγ-STAT1 and TNFα-NF-κB signaling pathways in triggering the transcription of AIM2 and PD-L1 in refractory OSCC. Consequently, AIM2 upregulation likely reprograms the tumor immune microenvironment towards an immune-inflamed condition characterized by increased IFNγ signaling and PD-L1 expression [35] and is reported to be more responsive to ICI treatment [36].

The IFNγ-related gene signature was recently found to be capable of selecting melanoma patients who probably benefited from ICI treatment [37]. In this study, the in silico analyses revealed that AIM2 upregulation is extremely associated with the activation of IFNγ-responsive pathway in the radioresistant OSCC samples and the infiltration levels of IFNγ-producing immune cells in TCGA HNSCC samples. It has been shown that IFNγ can trigger the transcription of the PD-L1 gene via activating STAT1 [28]. Nevertheless, without IFNγ supplement, the mutation of the STAT1- binding site within the PD-L1 promoter dramatically suppressed luciferase activity in the highly metastatic/radioresistant HSC3/HSC4 cells transfected with a plasmid containing PD-L1 promoter-driven firefly luciferase gene. In addition, the neutralization of IL-1β moderately inhibited the activity of STAT1 in the AIM2-overexpressing SAS cells. Moreover, recent reports demonstrated that IL-1β can activate the STAT1 pathway by negatively modulating ERK2 activation in the target cells [38] and synergistically elevates PD-L1 expression through the coordination with IFNγ in hepatocellular carcinoma [39]. Therefore, these findings implicate that AIM2 upregulation coordinates the activation of STAT1 and NF-κB by triggering the secretion of IL-1β through the AIM2 inflammasome machinery, thereby governing PD-L1 transcription in refractory OSCC.

TNFα plays a critical role in the onset of immune response and is produced mainly secreted by macrophages even though other immune and non-immune cells, e.g. fibroblast, can produce TNFα [40]. TNFα has been associated with the progression and prognosis of different cancer types by perturbing several signaling pathways, e.g., Erk1/2 and NF-κB [32]. Besides, TNFα coordinated with IFNγ to promote MUC16 (CA125) expression in breast and ovarian cancer via the NF-κB signaling axis [41]. Moreover, TNFα secreted from the infiltrating macrophages enhanced PD-L1 expression by activating NF-κB, resulting in a poor prognosis in pancreatic cancer [42]. Here we found that AIM2 upregulation is highly associated with the activation of the TNFα-NF-κB signaling axis, which is likely regulated by the infiltrated macrophages and constitutively elevates PD-L1 expression via IL-1β-NF-κB pathway in refractory OSCC. Therefore, these findings suggest that AIM2 expression could serve as a valuable biomarker for predicting the therapeutic effectiveness of TNFα and PD-L1-targeting agents and NF-B inhibitors in the refractory OSCC.

Although there were some ongoing clinical trials for ICIs in OSCC [43], the FDA approved nivolumab and pembrolizumab for patients with relapsed/metastatic HNSCC [44]. Here we find that AIM2 upregulation refers to a favorable progression-free survival rate in cancer patients receiving nivolumab, pembrolizumab, atezolizumab or ipilimumab monotherapy even though its upregulation was not significant for predicting the responsiveness of cancer patients who received nivolumab or atezolizumab monotherapy in ROC plot analysis. Therefore, our findings suggest that AIM2 might be a valuable biomarker for predicting the anti-cancer effectiveness of pembrolizumab and ipilimumab on the radioresistant/metastatic OSCC.

## Conclusion

AIM2 upregulation may constitutively activate the IL-1β-STAT1/NF-κB signaling loop to elevate PD-L1 expression and probably trigger the infiltration of IFNγ and TNFα-producing immune cells to reinforce the activity of STAT1 and NF-κB, respectively, in the refractory OSCC. Although further experiments are still needed to explore the role of AIM2 in promoting the reprogramming of tumor immune microenvironment, our results suggest that ICIs could be an effective therapeutic strategy to combat the refractory OSCC with AIM2 upregulation.

## Data Availability

Not applicable.

## Abbreviations

OSCC: Oral squamous cell carcinoma
HNSCC: Head and neck squamous cell carcinoma
ICIs: Immune checkpoint inhibitors
AIM2: Absent in melanoma 2
PD-L1: Programmed death-ligand 1
STAT1: Signal Transducer and Activator of Transcription 1
HPV: Human papillomavirus
IFNγ: Interferon-gamma
IL-1β: Interleukin-1β
EMT: Epithelial–mesenchymal transition
TCGA: The Cancer Genome Atlas
GEO: Gene Expression Omnibus
JCRB: Japanese Collection of Research Bioresources
RT-PCR: Reverse transcription-polymerase chain reaction
GAS: IFNγ activation site
GSEA: Gene Set Enrichment Analysis
